# Cytokine Networks and the Clinical Outcome of American Teg-Umentary Leishmaniasis: Unveiling Targets for Alternative Therapeutic Interventions

**DOI:** 10.3390/pathogens14020188

**Published:** 2025-02-13

**Authors:** Carolina Cattoni Koh, Kenneth J. Gollob, Walderez O. Dutra

**Affiliations:** 1 Department of Morphology, Institute of Biological Sciences, Federal University of Minas Gerais, Av. Pres. Antônio Carlos, 6627-Pampulha, Belo Horizonte 31270-901, MG, Brazil; 2 National Institute of Science and Technology in Tropical Diseases, INCT-DT, Salvador 40110-160, BA, Brazil; kenneth.gollob@einstein.br; 3 Hospital Israelita Albert Einstein, São Paulo 05652-900, SP, Brazil

**Keywords:** Leishmaniasis, immunoregulation, cytokines, therapy

## Abstract

American Tegumentary Leishmaniasis (ATL), caused by parasites of the genus *Leishmania*, presents a significant global health challenge, especially in Brazil, where cutaneous and mucosal forms are highly prevalent. Cutaneous Leishmaniasis (CL) typically results in single lesions, while mucosal Leishmaniasis (ML) leads to destructive mucosal lesions with a worse prognosis. The immune response, regulated by cytokines, plays a crucial role in disease progression and resolution. In CL, a balance between pro-inflammatory and anti-inflammatory cytokines is associated with lesion resolution, whereas in ML, an exaggerated inflammatory response worsens tissue damage. Thus, understanding cytokine regulation is essential for unveiling disease pathology and developing effective immunotherapeutic strategies. Here we discuss gene polymorphisms and epigenetic modifications that affect cytokine expression, influencing disease susceptibility and severity, as well as immunotherapeutic approaches that involve cytokine function in Leishmaniasis. In addition, we examine advancements in drug discovery, utilizing in silico methods and targeted drug delivery systems, providing potential avenues for better therapeutic interventions. Continuous research into immune responses and cytokine production and function is critical for identifying novel therapeutic targets and optimizing patient care for ATL.

## 1. Introduction

Human infection with parasites of the genus *Leishmania* results in a spectrum of diseases, including tegumentary and visceral forms, that affect millions of people globally. In Brazil, the most prevalent forms of Leishmaniasis include cutaneous and mucosal tegumentary forms [1]. According to the Ministry of Health, in 2022, Brazil reported over 12,000 cases of American Tegumentary Leishmaniasis (ATL), with more than 80% of cases occurring in individuals over 20 years old, and the majority affecting males [2]. While ATL is not fatal, its progression and treatment side-effects pose serious challenges for individuals in terms of work and leading a regular life [3], affecting the patient as well as their family and society.

Cutaneous Leishmaniasis (CL) involves the initial formation of a papule or nodule at the site of infection, progressing to an ulcerated lesion. This cutaneous manifestation typically occurs as a single lesion, although multiple lesions may appear. While in some cases CL lesions may spontaneously heal after a few weeks or months, the number of self-healing cases has been declining. A wide variety of *Leishmania* species can cause classic CL, particularly in the New World. These species include members of the *Leishmania (Leishmania)* subgenus, such as *L. (L.) amazonensis and L. (L.) mexicana, as well as the Viannia subgenus, represented by L. (V.) braziliensis, L. (V.) guyanensis, L. (V.) panamensis, and L. (V.) peruviana* [4]. Less commonly, ATL is caused by *L. (V.) shawi, L. (V.) naïffi, L. (V.) lainsoni, and L. (V.) lindenbergi* [4].

In Brazil, *Leishmania (Viannia) braziliensis* is the primary species associated with CL, though other species such as *L. (V.) guyanensis, L. (V.) panamensis, and L. (L.) amazonensis* are also implicated [5]. Notably, certain species have associations with distinct forms of the disease: *L. (V.) braziliensis, L. (V.) guyanensis, and L. (V.) panamensis* are linked to mucosal (ML), mucocutaneous (MCL), and disseminated Leishmaniasis (DL), while *L. (L.) amazonensis and L. (L.) mexicana* are capable of causing diffuse cutaneous Leishmaniasis (DCL) [4].

Mucosal Leishmaniasis (ML), predominantly caused by *Leishmania (Viannia) braziliensis* in Brazil, is characterized by disfiguring lesions in the mucosa, often appearing months or years after the resolution of the initial cutaneous lesions, whether by apparent spontaneous healing [6,7,8,9]. Its etiology results from the hematogenic or lymphatic dissemination of amastigotes to the mucosa of the oropharynx, larynx, nasal, and oral regions. Although the mechanisms underlying this migration remain poorly understood, ML is recognized as a delayed clinical condition linked to cutaneous infections by *L. (V) braziliensis* [6,7,8,9]. The pathogenesis of ML is closely associated with the host’s cell-mediated immune response, as evidenced by the scarcity of parasites in mucosal lesions. This underscores the dual role of the immune system in parasite elimination and in contributing to disease severity. ML generally does not exhibit spontaneous healing and is considered a progressive and destructive disease [8].

The treatment of ATL varies across different countries and regions, reflecting the complexity of therapeutic approaches. Various factors, such as different *Leishmania* species, susceptibility to drugs, disease manifestations, variations in the host’s immune response, and the pharmacokinetic properties of drugs, influence the efficacy and choice of therapy [10].

## 2. The Role of Cytokines in Different Clinical Forms of ATL

The less severe characteristic of CL lesions heavily relies on the initial immune response [11], and most of the initial findings have been described using experimental models of *Leishmania* infection. Upon infection, a variety of immune cells, including monocytes, dendritic cells (DCs), and neutrophils, are recruited to the infection site, where they phagocytize *Leishmania* parasites [11]. Neutrophils play dual roles during infection, serving as Trojan horses for the parasites and acting as leishmanicidal agents [12]. Additionally, it has been demonstrated that neutrophil extracellular traps (NETs) efficiently destroy promastigotes of *L. (L) amazonensis* [13]. Conversely, the phagocytosis of apoptotic neutrophils infected with *L. (L) major* prevents the activation of macrophages and DCs, prolonging the life of the parasites [14].

Interleukin (IL)-12 plays a pivotal role in controlling *Leishmania* infection by fostering the Th1 response and inducing the production of gamma interferon (IFN-γ) [15]. Peripheral blood mononuclear cells (PBMCs) from patients with acquired immunity to CL, stimulated with *L. (L) major* antigen, exhibit increased levels of IFN-γ and tumor necrosis factor (TNF)-α [16]. Reactive oxygen species (ROS) are produced in response to the activation of macrophages by TNF-α and IFN-γ, which contribute to eliminating *Leishmania* [17,18,19].

Reactive oxygen species (ROS) and nitric oxide (NO) are the two primary microbicidal mediators regulating *Leishmania* infection. Phagocytic cells can quickly mediate anti-parasitic activity in the early stages of infection, even in the absence of activation, through ROS production [18,19]. These molecules play a particularly crucial role in CL, especially in *L. (L) braziliensis* infections, as *L. (L) braziliensis* amastigotes are susceptible to ROS and thrive and replicate more readily in their absence [19]. After activation by IFN-γ and TNF-α, inducible nitric oxide synthase (iNOS) produces NO, leading to the death of *Leishmania* parasites [20].

In human CL, early disease is characterized by intense immune activation, expressed as regional lymphadenopathy [21,22]. This event is associated with the activation of CD4^+^ and CD8^+^ cells, which express adhesion molecules that drive their recruitment to lesion sites [23]. Despite this activation, parasite load is higher in early CL compared to more advanced disease [24] and treatment is not as effective in early CL [25].

While a Th1 response is essential for controlling the infection, Th2 cytokines promote tissue regeneration and reduce inflammation severity during lesion healing [26]. Increased IL-10 levels may counteract the effects of IFN-γ, as elevated IFN-γ levels can lead to tissue damage [27]. Additionally, IL-4 and IL-13 can induce arginase-1 (Arg-1), a Th2 enzyme involved in tissue repair through collagen synthesis [28,29]. In an experimental model, it has been observed that increased IL-4 production and Arg I expression are associated with a more severe form of the disease, characterized by larger lesions, prolonged disease duration, and reduced parasite clearance, as demonstrated in the study of Costa (2011), where BALB/c mice infected with antimony treatment-refractory isolates of *Leishmania (Viannia) braziliensis* developed severe lesions due to IL-4 production [30]. 

These findings imply that both Th1 and Th2 immune responses are essential for pathology control.

IL-17 aids in neutrophil recruitment and is involved in immunity to intracellular pathogens. Lesions from CL patients show the production of IL-17, indicating its role in supporting lesion formation and tissue damage, as well as in immunity. In *L. (V) braziliensis* infection, IL-17 and TNF-α production is reduced by IL-10 and transforming growth factor (TGF)-β, which can inhibit inflammation and tissue damage. Additionally, PBMCs from CL patients contain higher amounts of IL-17 than PBMCs from healthy donors [31].

Some patients with CL develop ML, influenced by factors such as the size and number of skin lesions, species and virulence of the parasite, host immune response, and age, sex, and nutritional status of the patient [32]. A hallmark of ML is elevated chronic inflammation with an exacerbation of the Th1 response. PBMCs from ML patients exhibit elevated production of IFN-γ and TNF-α and lower levels of IL-10 after antigenic stimulation compared to patients without mucosal involvement [33,34]. Furthermore, ML patients have lower amounts of regulatory monocytes producing IL-10 and a higher percentage of CD4^+^ T cells expressing IFN-γ and TNF-α in the blood [34,35].

As the disease progresses toward the more severe ML, the proportion of CD8^+^ T cells relative to CD4^+^ T cells increases. These CD8^+^ cells exhibit cytotoxic activity characterized by the expression of granzyme A [36]. In addition to CD4^+^ and CD8^+^ cells, ML lesions exhibit elevated levels of IFN-γ and a greater number of CD68^+^ macrophages [37]. Interestingly, in 2020, Koh and colleagues demonstrated that the presence of extracellular traps derived from CD8^+^ lymphocytes is associated with ML [38]. ML patients not only have an exaggerated Th1 profile but also show enhanced tissue destruction, a larger frequency of CD8^+^ cells producing granzyme A, and low expression of the IL-10 receptor in lesions, indicating yet another challenge in controlling and regulating the immune response [33,36,37]. 

Additionally, Ferraz and colleagues demonstrated in 2015 that during antimony treatment, patients exhibited reduced frequencies of total CD8^+^ T lymphocytes and increased levels of apoptosis in these cells, which were also observed following LbAg-stimulation culture [39]. High frequencies of effector CD8^+^ T cells were noted in patients during treatment, whereas lower frequencies were observed after treatment [40]. Interestingly, patients after treatment showed elevated frequencies of apoptotic-effector CD8^+^ T lymphocytes. Similar findings were observed in in vitro antigenic-stimulation assays. Correlation analysis indicated that larger lesion sizes correlated with decreased frequencies of effector CD8^+^ T lymphocytes in patients during and after treatment. Additionally, a positive correlation was found between the frequency of apoptotic-effector CD8^+^ T cells and lesion size in patients during treatment [40]. These results highlight and emphasize the importance of CD8^+^ T lymphocytes in lesion resolution. Novais and colleagues, in their 2013 study, discussed that cytolytic CD8^+^ T cells mediate immunopathology and drive the development of metastatic lesions in cutaneous Leishmaniasis [41].

**Figure 1:**
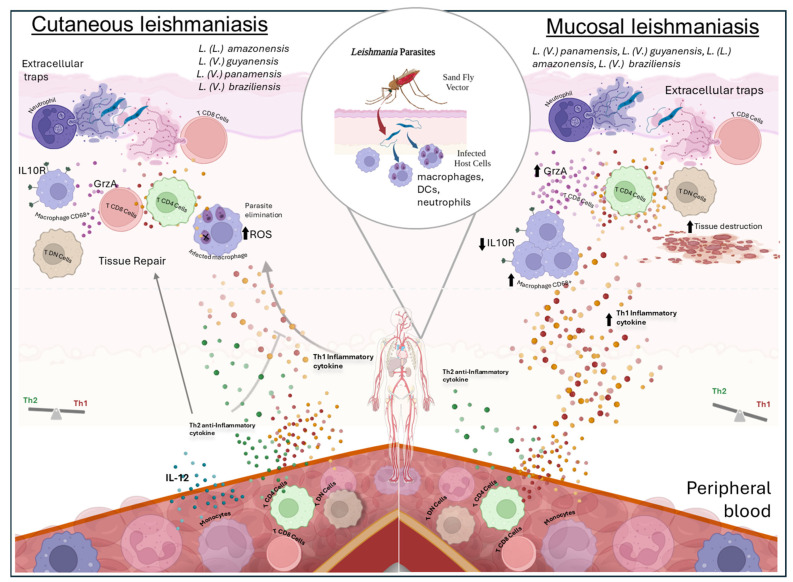
Leishmaniasis is a disease caused by parasites of the genus *Leishmania*.

Remarkably, it has been shown that senescent CD8^+^ T cells predominate in cutaneous lesions in Leishmaniasis [42]. Additionally, CD8^+^ T cells exhibit exhaustion in active cutaneous Leishmaniasis, whereas cured patients display a more robust multifunctional CD8^+^ T cell response and an increase in cells expressing a cytotoxic Th1-like profile (IFN-γ+/granzyme-B+/perforin+) compared to patients with active CL [43]. Figure 1 illustrates the relationship between clinical forms and cytokines.

Different species of *Leishmania* can trigger various forms of the disease, such as the cutaneous and mucosal forms. It is transmitted through the bite of the sand fly, leading to both local (skin and mucosal) and systemic manifestations (in peripheral blood). Following the sand fly bite, regurgitation of *Leishmania* promastigote forms occurs, which are then phagocytosed by tissue-resident cells such as macrophages, dendritic cells (DCs), and neutrophils. Despite the localized infection, alterations in cytokine production by cells in peripheral blood are observed. In this compartment, patients with the cutaneous form show increased production of IL-12 by monocytes, inducing lymphocytes to produce inflammatory cytokines such as IFN and TNF, as well as anti-inflammatory cytokines such as IL-10 and TGF-β. The production of these molecules is crucial for both controlling the inflammatory response and aiding tissue repair. TNF and IFN production stimulate infected macrophages in the skin to produce reactive oxygen species (ROS) that promote parasite elimination. In the skin infection site, extracellular traps of neutrophils (NETs) and lymphocytes (LETs) are observed in cutaneous patients. In mucosal Leishmaniasis, a higher occurrence of Th1 profile cytokines is observed in the peripheral blood of patients, while in the skin (lesion), there is a greater occurrence of inflammatory cytokines such as IFN, an increase in CD8 cells producing Granzyme A (GrzA), LETs, and a lower expression of IL-10 receptor (IL10R). This combination of immunological characteristics leads to increased tissue destruction.

## 3. Control of Cytokine Expression

Understanding the intricate mechanisms involved in cytokine expression control is essential, yet only a limited number of studies have explored this complex landscape in ATL. Biological strategies governing this regulation involve the influence of single nucleotide polymorphisms (SNPs), with alterations in transcription factors emerging as significant contributors. SNPs, characterized by changes in DNA occurring in at least 1% of a population, entail the substitution of one nucleotide for another and can manifest throughout the genome, spanning introns, exons, promoters, enhancers, or intergenic regions [44,45]. Specific gene polymorphisms have the potential to induce shifts in protein expression levels and functionalities, thereby being categorized as functional polymorphisms. Several studies were carried out to verify the occurrence of polymorphisms and the production of cytokines which are summarized in Table 1.

Epigenetics, defined by reversible modifications in gene expression without altering the DNA sequence, introduces an additional level of complexity to the cytokine expression control environment. Epigenetic modifications, including DNA methylation, histone modification, and non-coding RNA expression, play crucial roles in this process [60,61,62].

A class of non-coding RNAs (ncRNAs), most notably microRNAs (miRNAs), is involved in post-transcriptional regulation and is a key component of epigenetic processes. These endogenous small RNAs, called miRNAs, bind complexly to complementary mRNAs to influence mRNA degradation or disrupt protein translation [63,64,65].

The expression of cell miRNA can be influenced by parasite strain, isolation, load, and virulence factors, making studies challenging due to the considerable variety of variables [66]. Within the same *Leishmania* species, different strains can induce differential effects on miRNA expression [67].

In 2021, Souza and colleagues showed that miR-548d-3p was upregulated in *L. (V.) braziliensis*-infected THP-1 cells and in plasma from self-healed patients. This miRNA was predicted to target the chemokine pathway and inflammation, central to the pathogenesis of ATL [68]. Inhibition of miR-548d-3p reduced parasite growth and increased the production of CCL2, CCL5, and CXCL10 in infected THP-1 cells. The altered miRNA expression observed *in L. (V.) braziliensis*-infected THP-1 cells was also found in plasma samples from patients with active or self-healed CL. These miRNAs were predicted to target cytokine, chemokine, and signaling pathways, suggesting a link between the differentially expressed miRNAs and altered expression of these pathways [68].

In another investigation, patients with *L. (V) braziliensis* infection showed a correlation between certain miRNAs and accelerated wound healing [69]. Additionally, it was found that the expression of miR-361-3p, a regulator of GZMB and TNF, was much higher in lesions of ATL caused by *L. (V) braziliensis* compared to healthy skin. This increased expression was associated with both delayed healing time of cutaneous ulcers and therapeutic failure [70].

The miR-146a inhibits interleukin 1 receptor-associated kinase 1 (IRAK1) and tumor necrosis factor (TNF) receptor-associated factor 6 (TRAF6) in the toll-like receptor pathway, while miR-499a modulates TGF-β and TNF signaling pathways. A study by de Mesquita, in 2021, investigates the association of MIRNA146A rs2910164 and MIRNA499 rs3746444 variants with the development of *L. (V) guyanensis* (Lg)-cutaneous Leishmaniasis (CL) [71]. Results indicated that carriers of the rs2910164 CC genotype have a higher risk of developing CL, especially in males. Similarly, the G allele of MIR499A rs3746444 was associated with an increased risk of Lg-CL in males, with G allele carriers showing elevated odds of developing the disease. These findings suggest that both MIR146A rs2910164 and MIR499A rs3746444 are significantly associated with the development of Lg-CL, particularly in male individuals [71]. 

These intricate mechanisms emphasize the multifaceted regulation of cytokine expression, providing potential pathways for therapeutic interventions in diseases such as CL.

In addition to their role in the pathogenesis and progression of ATL, microRNAs have emerged as promising targets for innovative therapeutic strategies. miRNA mimics, such as those restoring miR-155 expression, could strengthen protective inflammatory responses [72]. Conversely, antagomiRs targeting miRNAs can suppress pathways that favor parasite survival [73]. Furthermore, the use of miRNAs as biomarkers represents a significant area of potential, enabling early detection of infections, evaluation of therapeutic responses, and prognosis prediction [74]. Growing evidence suggests that miRNA regulation plays a multifaceted role in the interaction between *Leishmania* and the host, emphasizing the need to integrate miRNA studies into future strategies to combat Leishmaniasis [75]. However, cytokine expression during *Leishmania* infection is not solely regulated by miRNAs. *Leishmania* employs multiple mechanisms, including the action of glycoconjugates and membrane-associated or secreted proteins, as well as the release of extracellular vesicles (EVs). These strategies influence phosphorylation cascades, affecting cellular activation and immune responses, which are critical for the parasite’s ability to evade the host immune system and drive disease progression [76,77]. Such mechanisms highlight the complexity of cytokine regulation during *Leishmania* infection and emphasize the multifaceted nature of host-parasite interactions.

## 4. Targeting Cytokine Modulation: Immunotherapeutic Strategies for Controlling ATL

The study of American tegumentary Leishmaniasis (ATL) faces significant challenges, particularly due to the scarcity of studies on human patients. Most research relies on animal models, which present limitations distinct from the challenges observed in human disease. Therefore, this review will highlight studies conducted on human patients, emphasizing their contributions to understanding the disease and addressing its unique clinical complexities. 

Pentavalent antimonial compounds, such as N-methylglucamine antimoniate (Glucantime), are recommended for treating cutaneous Leishmaniasis. While the exact structure and mechanism of action of antimonials (Sb) remain unknown, they likely involve the inhibition of adenosine triphosphate (ATP) and guanosine triphosphate (GTP) by disrupting the citric acid cycle and glycolysis. Additionally, antimonials may be activated and converted to the more toxic trivalent form (SbIII). The anti-Leishmanial activity of Sb may also result from the stimulation of host macrophages [78,79]. However, their use often leads to notable side effects, including nausea, vomiting, abdominal pain, and cardiotoxicity, highlighting the importance of careful evaluation of contraindications and clinical manifestations.

An alternative therapeutic option is Amphotericin B, which disrupts the parasite’s cell membrane by binding to ergosterols [80]. Despite its effectiveness, this treatment also carries its own set of side effects, emphasizing the critical need for patient selection based on clinical presentation and contraindications. Additionally, Pentamidine, which inhibits parasite DNA replication, emerges as a secondary option, especially in cases resistant to antimonials [81,82]

In 2010, the World Health Organization (WHO) Expert Committee on Leishmaniasis recommended the inclusion of local and topical treatments as acceptable therapeutic alternatives for New World Leishmaniasis [83]. Subsequently, in 2013, the Pan American Health Organization (PAHO) Expert Committee on Leishmaniasis incorporated intralesional treatment into the regional guidelines. However, this approach was restricted to reference centers and cases involving single lesions that do not affect the face or joints [84]. These guidelines were updated in 2022 to include more detailed therapeutic protocols for cutaneous, mucosal, and visceral Leishmaniasis, taking into account specific patient groups. The updates aim to expand access to treatment and improve clinical outcomes for patients across the Americas [85]. These recommendations reflect advancements in the management of Leishmaniasis, emphasizing the importance of less toxic and more accessible treatments, particularly in endemic areas [85].

Facing these challenges, the exploration of new drugs or treatment adjuvants becomes crucial. Recognizing the significance of cytokines in ATL′s progression and aggravation, the use of cytokines as immunotherapeutic strategies to control ATL becomes an alternative to traditional treatments. The use of Pentoxifylline as an immunomodulatory coadjuvant in ATL treatment and the inclusion of therapeutic regimens that lead to faster cure rates compared to conventional approaches have been demonstrated. Pentoxifylline acts as an adjuvant and is not recommended as a standalone medication but rather in conjunction with N-methylglucamine antimoniate [8,82,86,87,88,89]. Pentoxifylline is known for its ability to inhibit TNF-α [90], an inflammatory cytokine associated with more severe forms of ATL [34,35,89,91]. This is an example of how understanding cytokine involvement in disease leads to new and efficient therapeutic interventions.

Continuing to explore immunotherapies, the role of the Granulocyte-Macrophage Colony-Stimulating Factor (GM-CSF) was evaluated in ATL. GM-CSF plays essential roles in stimulating and recruiting key contributors to wound repair, such as keratinocytes, macrophages, and fibroblasts. Administered as a local injection, GM-CSF has shown a positive effect on chronic wound healing, accelerating the process by promoting the growth and migration of keratinocytes and aiding in reepithelialization, a critical step in wound closure [92]. Furthermore, GM-CSF enhances the recruitment and activation of macrophages involved in debris removal and tissue remodeling, and stimulates fibroblast proliferation and collagen synthesis, contributing to the formation of new tissues and effective wound closure [93]. Another study assessed the use of topically applied GM-CSF in combination with the standard dose of antimony to treat refractory cases of CL. Five patients enrolled in an open-label clinical trial, having undergone three or more courses of antimony, were treated with topically applied GM-CSF solution and standard parenteral antimony for 3 weeks. All patients healed their CL ulcers, with three achieving healing in 50 days and two in 118 and 120 days after the start of therapy. The combined treatment of topically applied GM-CSF and antimony proved effective and well-tolerated in treating CL relapse, with no reported side effects [94]. Thus, GM-CSF may play a promising role as part of immunotherapeutic strategies in the context of CL, contributing to the immune response and healing of skin lesions associated with *Leishmania* infection. 

Recent advancements in pharmaceutical research have leveraged in silico methods such as Virtual Screening to efficiently identify potential drug candidates while minimizing resource utilization. This approach, as highlighted by Piccirillo and Amaral (2018), elucidates the molecular mechanisms underlying the action of biologically active compounds and explores structure-activity relationships to enhance drug efficacy and safety [95]. Additionally, the utilization of site-specific drug delivery systems like liposomes and nanoparticles, as discussed by Quezada (2019) and Plaza Oliver (2021), offers promising avenues for enhancing treatment efficacy by reducing adverse effects and improving drug bioavailability [96,97]. These innovative strategies underscore ongoing efforts to advance drug discovery and development, particularly in addressing diseases such as ATL. 

However, in silico research has primarily focused on finding leishmanicidal drugs rather than emphasizing the immune response and cytokine production by the patient. A combined approach may emerge in the near future, allowing the design of drug/immunotherapy combinations to treat ATL.

## 5. Conclusions

The intricate relationship between cytokines and the immune response in different forms of ATL highlights the complexity of this disease. CL and ML exhibit unique immunological profiles influenced by cytokine expression, affecting disease progression. Genetic and epigenetic factors also impact cytokine regulation, influencing susceptibility and severity of ATL. Targeted immunotherapeutic approaches like Pentoxifylline and Granulocyte-Macrophage Colony-Stimulating Factor (GM-CSF) show promise in improving treatment outcomes. Advances in drug discovery and delivery methods offer hope for more effective and safer treatments. Continued research into immune responses and cytokine production is essential for identifying new therapeutic targets and enhancing patient care. Understanding cytokine regulation is key to shaping future ATL treatment strategies.

## Figures and Tables

**Table 1:** Cytokine-related Polymorphisms and Their Associations with Cutaneous and Mucosal Leishmaniasis.

Gene	Polymorphism	Main Findings	*Leishmania* Species	Population	Refs
*HLAG*	3′ UTR polymorphism 3142GC (rs1063320)	Association with ATL manifestations. *HLAG* polymorphisms may influence susceptibility and clinical manifestations of ATL. HLA-G can inhibit the immune response, affecting cytotoxic T cells, NK cells, and inflammatory cytokine production.	Not specified	Brazilian	[46]
*IFNG*	+874T/A	Not associated with susceptibility or severity. In CL, AA genotype produces less IFN-γ compared to TA/TT	*L. (V.) braziliensis.*	Brazilian	[47]
*IL10*	-819C/T (CC)	CC genotype more prevalent in CL patients	*L. (V.) panamensis*	Colombian	[48]
*IL13*	Various variants	Three haplotypes (CCCTAAC, ACCCGCT, AGCTAAC) associated with resistance and three haplotypes (ACCTGCC, ACCCAAT, AGCCGCC) associated with susceptibility to CL	*L. (V.) guyanensis*	Brazilian	[49]
*IL17A*	rs2275913 (G/A)	Significant association with susceptibility to CL	*L. (V.) braziliensis.*	Brazilian	[50]
*IL1B*	rs16944 T/C	Associated with CL	*L. (V.) guyanensis*	Brazilian	[51]
*IL2*	rs4833248, -2425G/A	No significant association found with susceptibility to CL	*L. (V.) guyanensis*	Brazilian	[52]
*IL2RA*	rs10905669, rs706778	Greater susceptibility to developing cutaneous lesions	*L. (V.) braziliensis.* and *L. (L.) tropica*	Brazilian and Iranian	[53]
*IL2RB*	rs2069762, +10558G/A	No significant association found with susceptibility to CL	*L. (V.) guyanensis*	Brazilian	[52]
*IL6*	IL6 Promoter -174 G/C	C allele associated with higher risk of ML. Lower IL-6 levels in CC genotype compared to GG	*L. (V.) braziliensis.*	Brazilian	[54]
*JAK3*	rs1003694, +13295T/C	No significant association found with susceptibility to CL	*L. (V.) guyanensis*	Brazilian	[52]
*MIF*	MIF-173C	*MIF* polymorphism is indeed associated with CL. MIF is a pleiotropic cytokine capable of inducing the production of other cytokines, such as TNF, IFN-γ, IL-1β, IL-12, and IL-6.	*L. (V.) braziliensis.*	Brazilian	[55]
*NOD2*	R702W, G908R, L1007FSinSC	Not associated with susceptibility to CL. Associated with low levels of IFN-γ, TNF-α, IL-17, and IL-8	*L. (V.) guyanensis*	Brazilian	[56]
*TGFB1*	Codon 25 (G/C)	G allele associated with resistance, while G/C and C/C genotypes indicate susceptibility to CL.	Not specified	Brazilian	[57]
*TLR4*	Various SNPs	No differences in allele frequencies for *TLR4* SNPs were observed. TLR-ligand interactions trigger signaling pathways that lead to the transcription of inflammatory mediators like IL-10 and TNFα	*L. (V.) panamensis*	Colombian	[48]
*TNFA*	-308g/A	A allele more common in asymptomatic individuals	*L. (V.) panamensis*	Colombian	[48]
	-308G>A	Individuals with A allele have higher susceptibility to leishmaniasis	Not specified	Varied (meta-analysis)	[58]
*TOLLIP*	rs5743899	Increased risk of developing CL. Regulates Toll-like receptor signaling pathways, impacting IL-6 and TNF-α production	*L. (V.) guyanensis*	Brazilian	[59]
